# High-Speed Erosion Behavior of Hydrophobic Micro/Nanostructured Titanium Surfaces

**DOI:** 10.3390/nano12050880

**Published:** 2022-03-07

**Authors:** Yong Chen, Jiguo Zhang

**Affiliations:** School of Mechanical Engineering, Shanghai Jiao Tong University, Shanghai 200240, China

**Keywords:** photoetching, titanium alloy, hydrophobicity, erosion test

## Abstract

Ice accretion on aircrafts or their engines can cause serious problems and even accidents. Traditional anti-icing and de-icing systems reduce engine efficiency, which can be improved by the use of hydrophobic/icephobic coatings or surfaces that reduce the amount of bleed air or electric power needed. These hydrophobic/icephobic coatings or surfaces are eroded by high-speed air flow, water droplets, ice crystals, sand, and volcanic ash, resulting in the degradation, material loss, or deterioration of the coating’s waterproof and anti-icing properties. Thus, the durability of hydrophobic micro/nanostructured surfaces is a major concern in aircraft applications. However, the mechanism responsible for material loss in hydrophobic micro/nanostructured surfaces resulting from high-speed erosion remains unclear. In this paper, hydrophobic titanium alloy surfaces with cubic pit arrays are fabricated by photoetching and tested using a high-speed sand erosion rig. Under the same impact conditions, the erosion rates of the micro/nanostructured titanium surfaces were similar to those of smooth titanium alloy, implying that the hydrophobic surface fabricated on the bulk material had erosion-resistant capabilities. The material loss mechanisms of the micro/nanostructures under different impact angles were compared, providing useful information for the future optimization of micro/nanostructures with the goal of improved erosion resistance.

## 1. Introduction

When an aircraft flies under icing weather conditions at a subsonic speed less than a critical Mach number, the windward surfaces of some components freeze due to the impact and accumulation of water droplets in the atmosphere. A large number of accidents and studies indicated that the accumulation of even a small amount of ice on key parts of the aircraft can decrease lift and increase midair drag, resulting in reduced aerodynamic quality (e.g., deteriorated maneuverability and stability) [1]. Icing on aircraft engines is an even more prominent issue than icing on the aircraft itself. Due to the high-speed rotation of the engine, the air in the inlet exists in a suction state, airflow is accelerated, and the static temperature is lowered; thus, the air-intake system is more susceptible to icing and is more likely to freeze. Under certain temperatures and humidities on the ground and in the air, the nacelle and strut easily encounter icing problems. Icing results in the distortion of the inlet flow field along with deteriorated engine performance. Furthermore, the shedding ice may damage the fan blade. Aircraft icing is a serious problem that jeopardizes flight safety, and icing-related flight accidents occur every year. According to the National Transportation Safety Board, 264 flight accidents were caused by icing between 1998 and 2007, including 94 major accidents resulting in 202 deaths. In 2004, a CRJ200 aircraft operated by China Eastern Airlines crashed in Baotou, China, as a result of deteriorated aerodynamic performance caused by wing frost (ice). 

In anti-icing and de-icing systems for aircraft and turbofan engines, electric heating or hot air (or hot oil) are usually used, reducing the efficiency of the engine. The adhesive strength of ice can be markedly reduced by applying hydrophobic or icephobic coatings or surfaces on components that ice easily, reducing the amount of bleed air or electric power needed and thus improving engine efficiency. 

Many researchers have contributed to the design, fabrication, and testing of hydrophobic surfaces. Abdulhussein et al. [2] summarized the advances in the fabrication of manmade superhydrophobic surfaces. Zhang et al. [3] reviewed the fabrication and characterization of superhydrophobic surfaces along with their emerging energy-related applications based on recent progress in research and development in this field. Celia et al. [4] reviewed recent progress in the preparation of manmade superhydrophobic surfaces. The mechanical wear robustness and long-term durability of most superhydrophobic surfaces are limited, restricting the use of superhydrophobic coatings in industrial applications. Milionis et al. [5] summarized the progress in the fabrication, design, and understanding of mechanically durable superhydrophobic surfaces. In that paper, the most successful approaches to producing robust surfaces that maintain their non-wetting states after wear or abrasive action are summarized for different categories of mechanical wear (substrate adhesion, tangential surface abrasion, and dynamic impact to ultrasonic processing underwater), and various recommendations for improving and quantitatively evaluating mechanical wear durability are discussed along with potential future research directions with the goal of developing more systematic testing methods that are acceptable for the industry.

Tang [6] fabricated spike-shaped microstructured arrays on brass substrates and obtained superhydrophobic metal surfaces using pulsed ultraviolet (UV) laser ablation. Hribar et al. [7] studied the influence of the processing parameters on the laser-ablation of stainless steel and brass during the engraving by nanosecond fiber laser. Yang et al. [8] confirmed that the surface chemistry is not the only factor to determine surface super-hydrophobicity. The laser-induced super-hydrophobicity was attributed to the synergistic effect of surface topography and surface chemical compositions. Mohazzab et al. [9] studied the laser ablation of titanium in liquid using a fiber laser. Zhai et al. [10] improved the bonding strength between environmental barrier coatings (EBCs) and silicon carbide fiber reinforced silicon carbide (SiC/SiC) by preparing microstructures on an SiC/SiC surface with a femtosecond laser. Min et al. [11] prepared superhydrophobic surfaces on Al substrates using a simple etching method and studied the optimal conditions for superhydrophobicity. Lu et al. [12] prepared superhydrophobic surfaces on hydrophilic titanium substrates using a simple and inexpensive electrochemical method that is expected to be easily adaptable for use in large-scale industry applications. Stability and friction tests indicated that the prepared titanium surfaces were robust.

Ghalmi et al. [13] evaluated the effects of aluminum anodization time on surface roughness along with the effects of polytetrafluoroethylene (PTFE) nanostructural roughness on ice adhesion strength via centrifuge adhesion tests in a cold chamber. The wettability characteristics were also evaluated using a goniometer at ambient temperature. The ice adhesion strength on PTFE-coated aluminum was lower after anodization for 90 min compared to after anodization for 20, 32, and 60 min. Susoff et al. [14] determined the adhesion strengths between different coatings and ice using 0° cone tests. The effect of surface roughness on ice adhesion was also examined. Applying a fluorine-containing coating to aluminum pins considerably reduced the shear strength between ice and the pins, and rough surfaces showed higher ice adhesion strengths than smooth ones. Fortin et al. [15] developed the centrifuge adhesion test to measure the icephobicities of several kinds of icephobic coatings. A version of the adhesion reduction factor was introduced to evaluate coating icephobicity, and the confidence level of the test results reached 95%. 

Kreeger et al. [16] proposed basic requirements for icephobic coatings and materials. The first requirement is that the coating must withstand erosion and wear along with other weathering conditions in terms of its structural integrity. In the erosion test, the velocity must be high enough to be analogous to the conditions encountered during flight. Caroline et al. [17] studied the properties of icephobic coatings for aerospace applications along with the effects of aging (weathering and erosion) and the number of ice/de-icing cycles on coating durability. To identify icephobic coatings for aerospace applications, the authors proposed a selection process involving the following steps: the screening of candidate coatings with the ability to decrease ice adhesion; the evaluation of candidate coatings on a scaled-down two-blade rotor; and sand and rain erosion testing to evaluate the physical resistance of the coating. The durabilities of the most promising icephobic coatings were evaluated under rain and sand erosion as well as through exposure to multiple icing/de-icing cycles. Janjua et al. [18] evaluated the anti-icing performance of several kinds of coatings and assessed the durability of coating performance over multiple cycles of glaze ice growth and detachment from the coated substrate. The results show that the initially strong icephobic performance deteriorates quickly. Golovin et al. [19] studied the mechanisms responsible for the extremely low ice adhesion between coatings and ice obtained by tailoring the cross-link densities of different elastomeric coatings and enabling interfacial slippage. Golovin et al. fabricated and tested extremely durable coatings that maintained ice/coating shear strength less than 10 kPa after severe mechanical abrasion, acid/base exposure, 100 icing/de-icing cycles, thermal cycling, and accelerated corrosion. Cerro et al. [20] used ultra-short pulsed lasers to generate patterns on the surfaces of hard materials with the micrometer-scale features required for achieving superhydrophobicity. These surfaces demonstrated higher structural strength and lower deterioration rates compared to typical superhydrophobic coatings. Cerro et al. investigated the anti-ice properties of plasma-deposited hard coatings (e.g., diamond-like carbon) in combination with laser-machined patterns. These hard coatings exhibit reduced the surface energy and adjustable surface topography, which improve the erosion resistance of superhydrophobic surfaces and make them more suitable for use in harsh environmental conditions. Gutiérrez-Fernández et al. [21] reported on the simple fabrication of a library of ordered nanostructures by repeated irradiation using a nanosecond pulsed laser operating in the UV and visible region in order to obtain nanoscale-controlled functionality. Raimondo et al. [22] studied the amphiphobic behavior and durability of engineered aluminum alloy surfaces. Zhang et al. [23] studied the droplet erosion durability of a commercially available superhydrophobic coating. The hydrophobicity and icephobicity characteristics of the coating were compared before and after the droplet erosion experiment, and the level of damage to the coating was evaluated. The main researches are summarized in Table 1.

In actual applications, hydrophobic and icephobic coatings and surfaces are eroded by high-speed air, water droplets, ice crystals, sand, and other materials, resulting in the degradation, loss, or even deterioration of the waterproof and anti-icing functions. However, the mechanism of erosion damage to hydrophobic and icephobic coatings remains unclear. In this study, hydrophobic titanium alloy surfaces with cubic pit arrays were fabricated by photoetching and tested using a high-speed sand erosion rig. The erosion rates and microscale morphologies of the eroded surfaces were investigated, and the material loss mechanisms of the micro/nanostructures under different impact angles were compared and analyzed. The findings provide useful information for the future optimization of micro/nanostructures with the goal of improving erosion resistance.

## 2. Experimental Procedures

### 2.1. Fabrication of the Microscale Binary Hydrophobic Surfaces

The following materials were used: a round Ti-6Al-4V alloy sheet with a thickness of 2 mm and a diameter of 76.2 mm made by Baoji Youshengdee metal material Co., Ltd (Baoji, China); 19% hydrochloric acid solution; 34% nitric acid solution; a photoresist; a mask; acetone; and ethyl alcohol.

**Figure 1:**
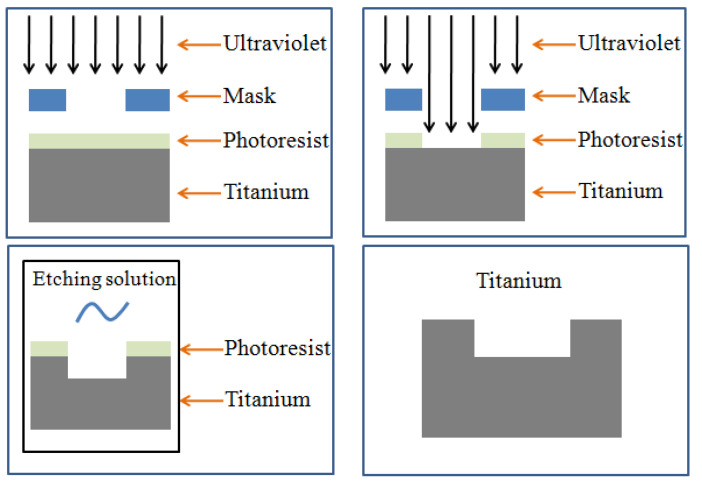
Schematic of the photoetching process.

Photoetching is a combination of graphic etching and chemical etching. The photoetching process is shown in Figure 1. Positive photoresist processing flow was adopted in this study. The detailed steps of this process were as follows: (1) the surface of the titanium alloy substrate was cleaned; (2) the photoresist was evenly coated on the surface of the titanium alloy; (3) a SUSS MA6/BA6 UV photolithography provided by SUSS MicroTec Group (Garching, Germany)were used to engrave a square array with the distance of 20 μm on the photoresist; (4) the exposed titanium alloy was dipped in the developing solution; (5) hydrochloric acid solution and nitric acid solution were mixed (volume ratio of hydrochloric acid to nitric acid = 3:1) as a corrosion agent, and the titanium alloy with the square array pattern photoresist was corroded for 2 min in a corrosion agent; and (6) the photoresist was removed, and the titanium alloy surface was cleaned by acetone and dried.

**Figure 2:**
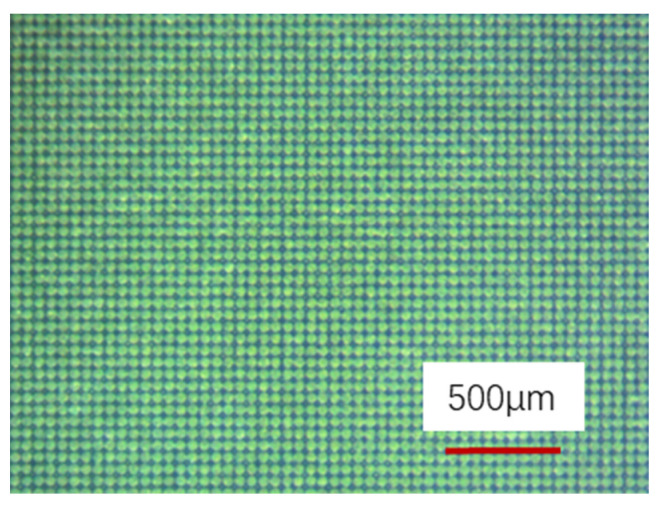
Optimal microscopy image of the photoetched titanium surface.

Figure 2 shows the photoetched titanium surface under optical microscope. Regular micron-sized pit arrays were distributed on the surface, which matched the pre-designed mask very well. The pits on the titanium surface were round rather than the expected square shape due to the isotropic corrosion caused by the etching solution.

### 2.2. Roughness and Contact Angle Measurements

The surface roughness of the etched titanium alloy was evaluated using a three-dimensional surface optical profilometer. The contact angles (CAs) of the surface of the smooth titanium alloy, the corrosion-treated surface, and the photoetched surface were measured using a surface CA measuring instrument. The detailed results are presented and discussed in Section 3.

### 2.3. Sand Erosion Test

**Figure 3:**
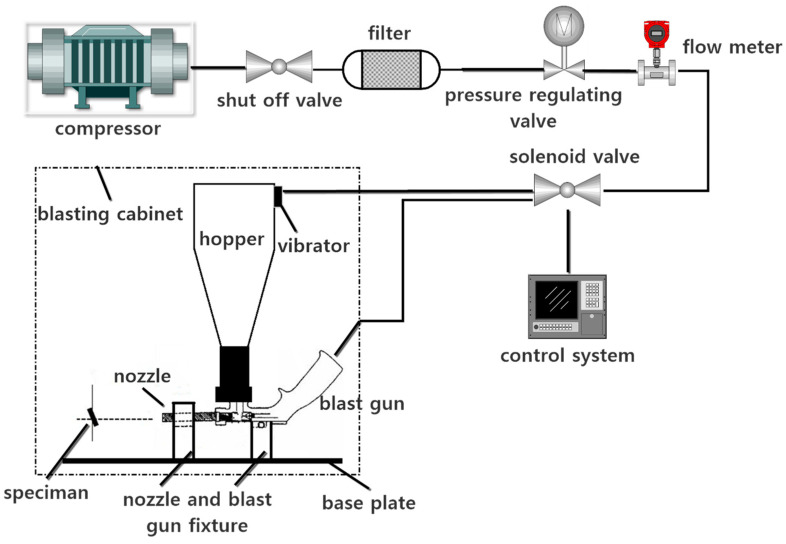
Schematic diagram of the erosion rig.

**Figure 4:**
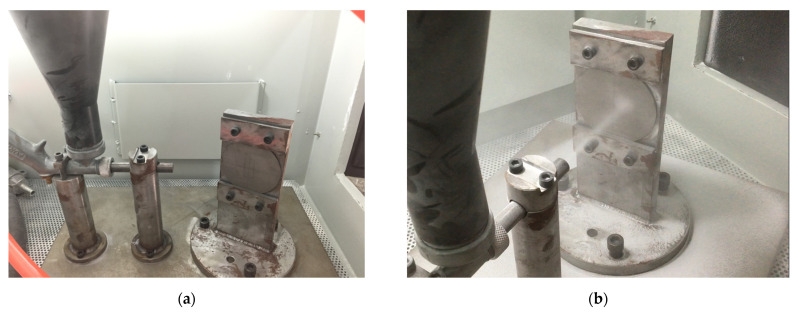
Photographs of the assembled erosion test rig. (**a**) Photo of the blast gun, hopper, and specimen. (**b**) Photo taken during an erosion test.

The high-speed sand erosion test rig used in this study was designed based on the ASTM G76-18 standard. A simplified schematic of the erosion rig is shown in Figure 3. The erosion rig consists primarily of a gas source, sandblasting system, measuring system, control system, and other auxiliary systems. The main components are an air source, regulating valve, air flow switch, pressure gauge, erosion device, and dust removal device. The airflow can be changed by adjusting the pressure regulating valve. An image of the assembled erosion rig is shown in Figure 4. The air source was connected to the bottom of the sandblasting gun. The hopper and the sandblasting gun were connected by the threaded holes on the sandblasting gun, allowing the erosion sand to enter the sandblasting gun from the hopper. The sand impact speed could be changed by adjusting the inlet air pressure. After acceleration in the nozzle, the sands impacted the test piece at the desired speed. The erosion angle could be changed by adjusting the fixed position between the clamp and the bottom plate. The impact speed range of the erosion rig was 30–250 m/s. The diameters of the erosion particles ranged from 40 to 100 μm. The other parameters are shown in Table 2.

### 2.4. Pre-Experimental Erosion Test Bench Calibration

**Figure 5:**
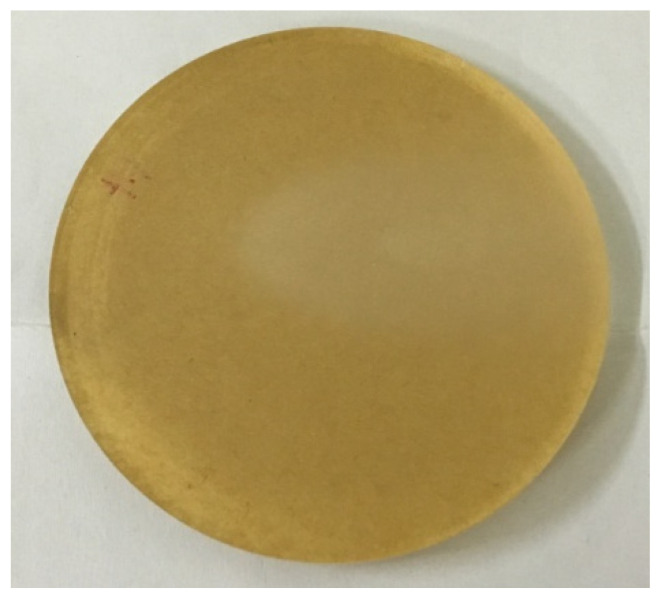
Image of the poly (methyl methacrylate) specimen for calibration of the erosion test rig.

The calibration of the erosion test rig is critical to ensure that the test parameters remain constant in each experiment. A poly (methyl methacrylate) specimen (Figure 5) was used for calibration before each test. During each calibration test, the impact angle was adjusted to 20°, and the inlet air pressure was set to 0.26 MPa. The calibration test results were evaluated by comparing the erosion position, erosion area, erosion depth, and material loss. If the difference between the erosion depth and material loss was within 10%, the test bench can be applied in the following erosion test. This step is critical because the high-hardness tungsten carbide nozzle can be severely worn. This can cause the inner diameter of the nozzle to increase, altering the erosion speed, erosion area, erosion depth, and material loss. Thus, the erosion rig must be calibrated before erosion testing.

## 3. Results

### 3.1. Surface Roughness and Contact Angle

**Figure 6:**
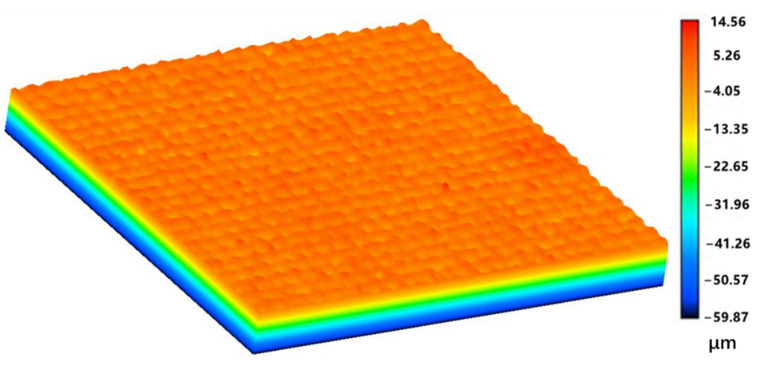
Surface morphology of the titanium surface treated by photoetching.

The morphology of the photoetched surface depends on the size and density of the photo-mask and the duration of acid corrosion. A three-dimensional surface profiler was used to measure the surface roughness of the treated surface. Figure 6 shows a 1000 × 1000 μm area of the surface. The average depth of the concave cavity corroded by the acid solution was approximately 3 μm, and the micro-scale roughness can be clearly observed in Figure 6, which was approximately 3 μm. 

**Figure 7:**
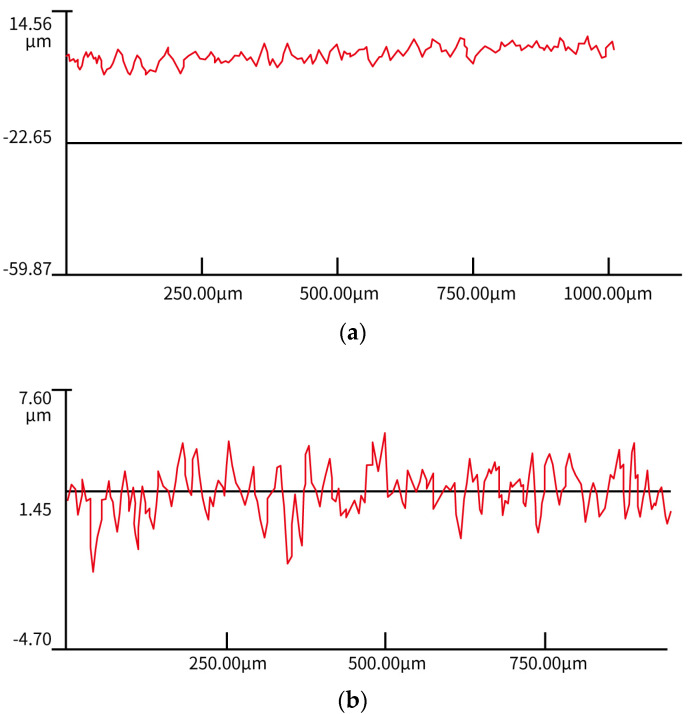
Comparison of the surface roughness between titanium surfaces treated by photoetching and corrosion. (**a**) Roughness of the titanium surface treated by photoetching. (**b**) Roughness of the titanium surface treated by direct corrosion.

**Figure 8:**
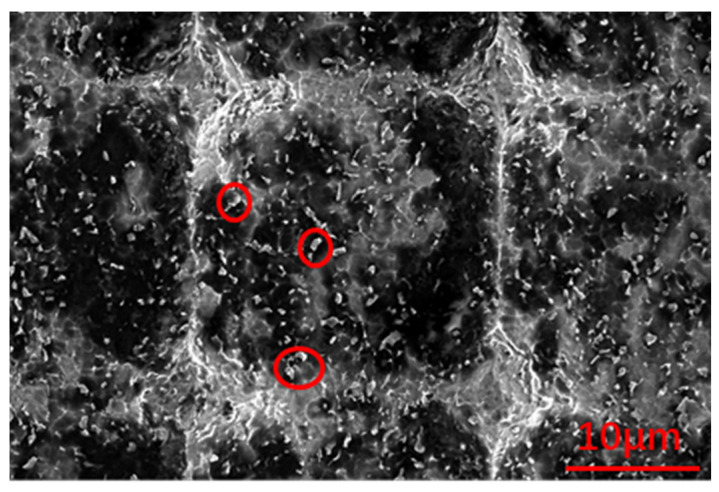
SEM image of a photoetched surface.

Figure 7 shows the roughnesses of the titanium surfaces treated by photoetching and corrosion, respectively. Comparing the microstructure and three-dimensional roughnesses of two different locations on the titanium surface indicates that the photoetched surface had a more regular microstructure than the directly corroded titanium alloy surface. Figure 7a indicates that nanoscale roughness was created on the surface during the photoetching process. The SEM image of the photoetched surface (Figure 8) clearly shows many small, raised zones (marked by red circle). This microstructure would benefit the hydrophobicity of the surface.

**Figure 9:**
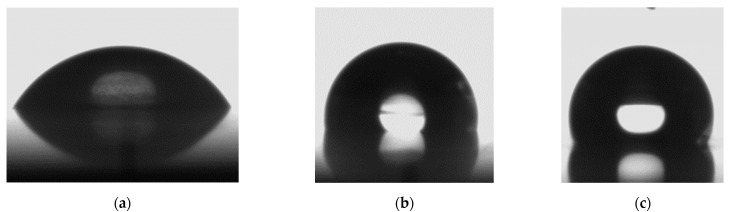
Images of water droplets on different surfaces during CA measurement. (**a**) Smooth surface. (**b**) Corroded surface. (**c**) Photoetched surface.

Figure 9 shows the CAs measured on different titanium surfaces. The CA of distilled water on the smooth and fresh titanium surface without any treatment is 60.2°, as shown in Figure 9a. The CA was increased to 100° after the titanium surface was corroded with the mixture of hydrochloric acid and nitric acid for 2 min (Figure 9b). Photoetching the smooth titanium surface increased the CA to 114.6°, indicating the generation of a hydrophobic surface (Figure 9c). The change in wettability is related to the microstructure of the surface. The acid corrosion of the smooth titanium surface created a nano-topography on the surface, which increased the roughness and the CA. The photoetched titanium surface exhibited a microscale binary array structure that caused the water droplet on the surface to exist in a Cassie–Baxter state, resulting in hydrophobic capability. The wettability of the surface could be improved by optimizing the micro/nanostructure. For example, the depth of the hemispherical concave features could be increased. However, this would increase the length of the standing wall, which behaves similar to a cantilever beam, thereby reducing the anti-erosion ability of the surface. Thus, the appropriate balance should be struck between wettability and anti-erosion ability.

### 3.2. Macroscale Morphology and Erosion Rate

**Figure 10:**
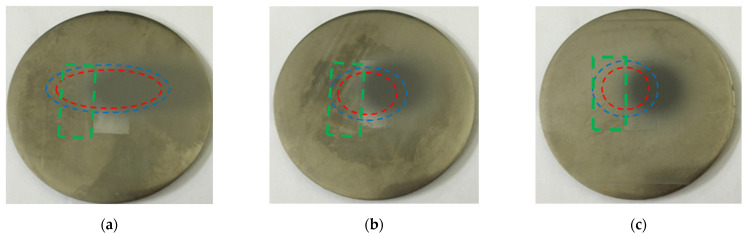
Images showing the macroscale morphologies of the titanium specimens eroded at different erosion angles. The green rectangles, red dotted lines, and blue dotted lines indicate the hydrophobic areas, seriously eroded areas, and transition areas, respectively. (**a**) 30°; (**b**) 60°; (**c**) 90°.

Figure 10 shows the macroscale morphologies of the eroded titanium surfaces with binary micro/nanostructures formed with erosion angles of 30°, 60°, and 90°. In Figure 10, the hydrophobic areas are marked by green rectangles, the seriously eroded areas are enclosed by red dotted lines, and the transition areas are marked by blue dotted lines. The area of the erosion zone increased with increasing erosion angle.

The erosion rate, q, is defined as the loss of specimen mass, $m_{e}$ (g), over total specimen mass, $m_{p}$ (kg), during the entire test process:

(1) $$q=\frac{m_{e}}{m_{p}}$$

**Figure 11:**
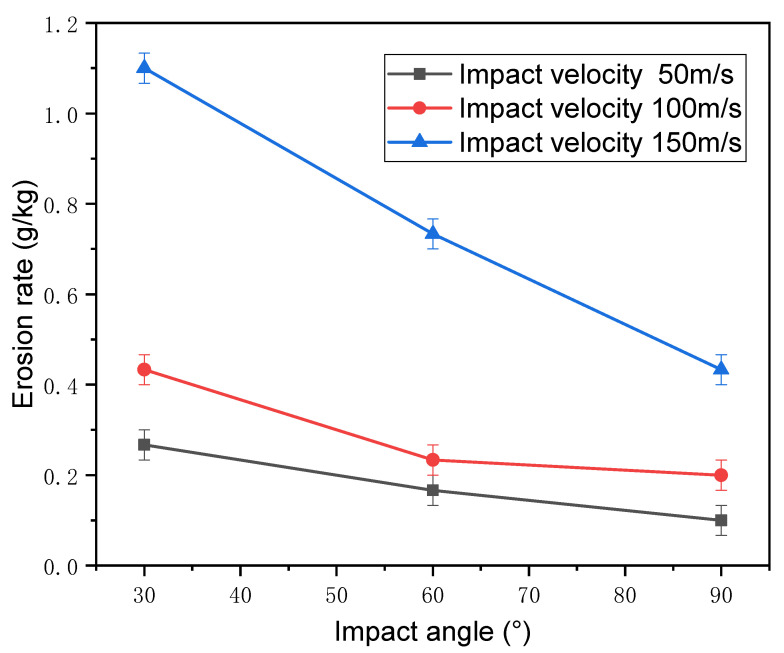
Erosion rate as a function of the impact angle under different impact velocities.

The relationship between the erosion rate and erosion angle is shown in Figure 11. For the same impact velocity, the erosion rate decreased with the increasing erosion angle.

The erosion rate at the erosion angle of 30° was larger than those at 60° and 90°. At the angle of 30°, the erosion particles quickly rubbed against the surface of the titanium alloy, cutting the surface. Under this condition, the main form of erosion failure was micro-cutting. When the erosion particles contacted the titanium surface at a low impinging angle, plowing force and squeezing force acting on the surface were the main causes of micro-cutting. 

At the large erosion angle of 90°, the main failure modes were forging extrusion and deformation wear. Titanium alloy has good plasticity, and cyclic hardening occurs under the impact of a large number of particles. Material loss occurs when the impact number is sufficiently large and the impact stress is higher than the yield strength of the material. On the other hand, plastic deformation squeezes the surface of the material to form a lip around the impact site, and subsequent erosion particles impact the lip until the material breaks off the surface, resulting in erosion loss. Comparing the erosion rates and erosion mechanisms under different erosion angles clearly shows that the erosion damage generated at low erosion angle was more serious, and the erosion resistance was worse compared to higher erosion angles. 

**Figure 12:**
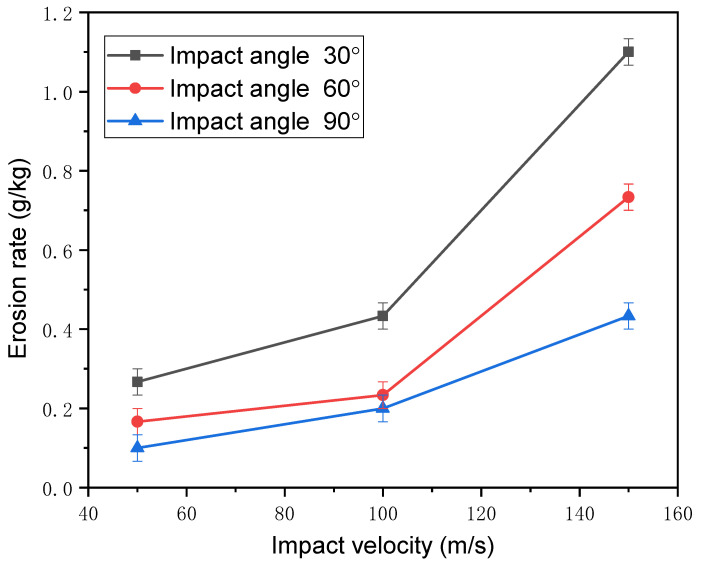
Erosion rate as a function of impact velocity under different impact angles.

As the impact velocity increases, the erosion rate of the titanium alloy also increases, as shown in Figure 12. When the impact speed was low, the kinetic energy carried by the particles was also low. Under this condition, the plowing force, pressing force, and impact force generated after the particles contacted the surface of the titanium alloy were small. Both the deformation loss and the forging extrusion loss were small; thus, the total amount of material loss was relatively small, and the erosion rate was low. As the erosion velocity increased, the kinetic energy of the particles increased, and a large plowing force, pressing force, and impact force were generated after the particles contacted the surface of the titanium alloy. In this case, the total amount of erosion loss and the erosion rate was greater compared to a lower impact velocity.

**Figure 13:**
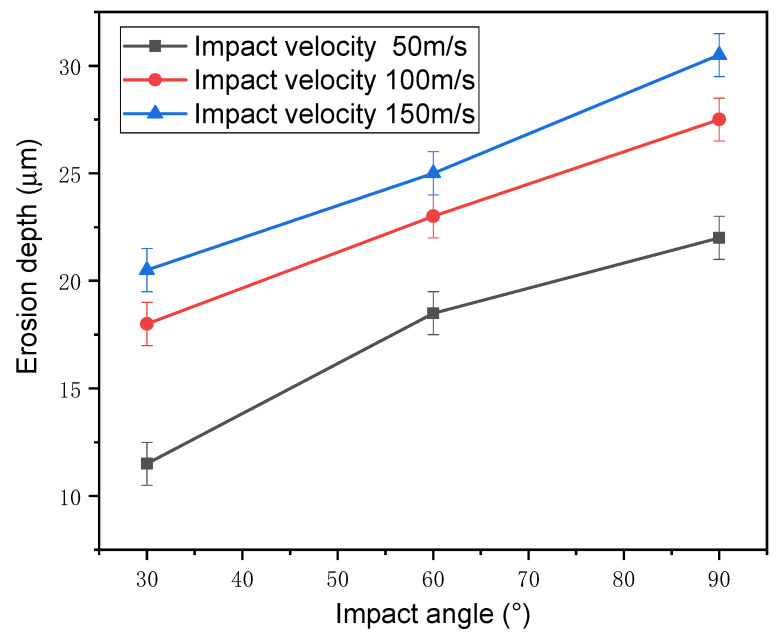
Erosion depth of the hydrophobic titanium surface as a function of impact angle for different impact velocities.

A three-dimensional optical surface profilometer was used to measure the surface roughness and erosion depth. The relationship between the erosion depth, erosion velocity, and erosion angle is shown in Figure 13. As the erosion angle increased, the erosion depth gradually increased. Although the total erosion wear was larger under a smaller erosion angle, the erosion area was much larger compared to under a high erosion angle. Under a large erosion angle, the total erosion wear of the hydrophobic titanium surface was small, the erosion area was small, and the erosion particles were more concentrated; thus, the maximum depth of the erosion pit was larger than that observed under a small erosion angle.

**Figure 14:**
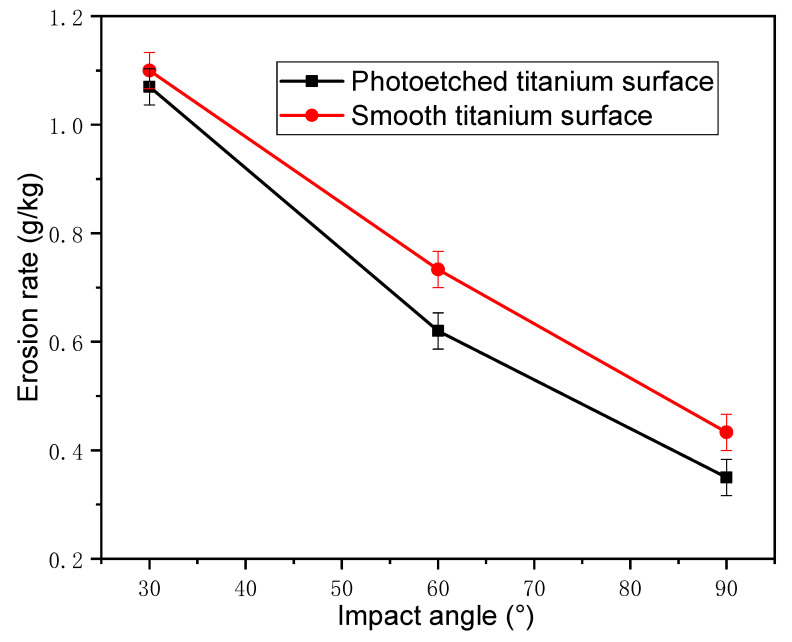
Erosion rates of the smooth and photoetched titanium surfaces as functions of the impact angle.

Figure 14 shows the erosion rates of the smooth and hydrophobic titanium alloy surfaces under an erosion speed of 150 m/s and different erosion angles (30°, 60°, and 90°). Compared to the untreated smooth alloy surface, the photoetched hydrophobic surface had a slightly higher erosion rate. Although the micro/nanostructure of the hydrophobic surface reduced its erosion resistance, its erosion rate was only slightly lower than that of the smooth titanium alloy surface. This is because the depth and width of the structure were small, indicating that the hydrophobic surface fabricated on the bulk material had erosion-resistant capability.

### 3.3. Microscale Morphology of the Eroded Surface

**Figure 15:**
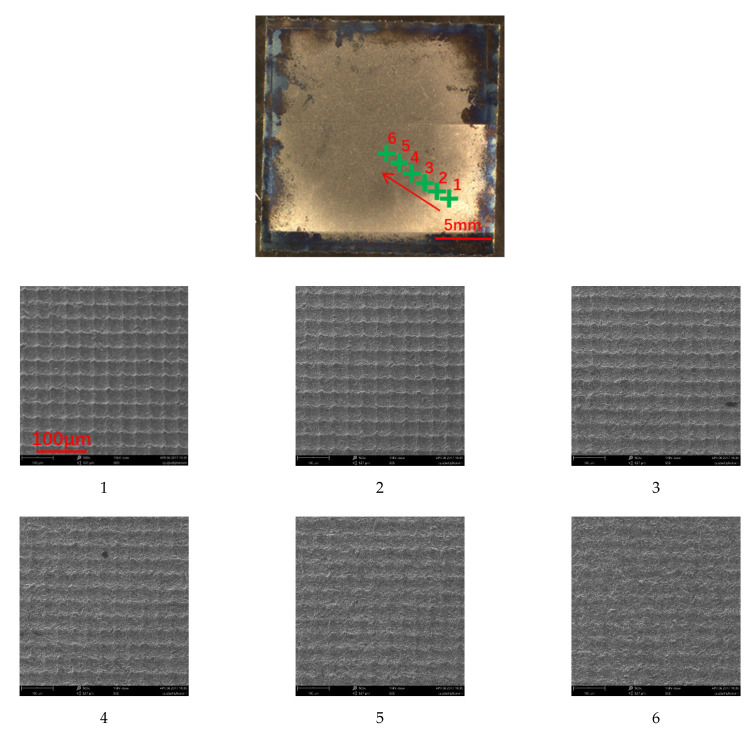
Microstructures of the titanium plate at six different positions.

Figure 15 shows the SEM images depicting the microstructures of the titanium plate at six different positions spanning the undamaged area to the heavily eroded area. The micro/nanostructures on the titanium surface were gradually damaged from the edges of the eroded area to the middle of the eroded area. The difference in the erosion depth on the titanium surface resulted from the expansion of the jet flow sprayed from the nozzle, which caused the particle speed in the core area to be higher than that in the transition and edge areas. In the core eroded area, the micro/nanostructures were almost completely damaged.

**Figure 16:**
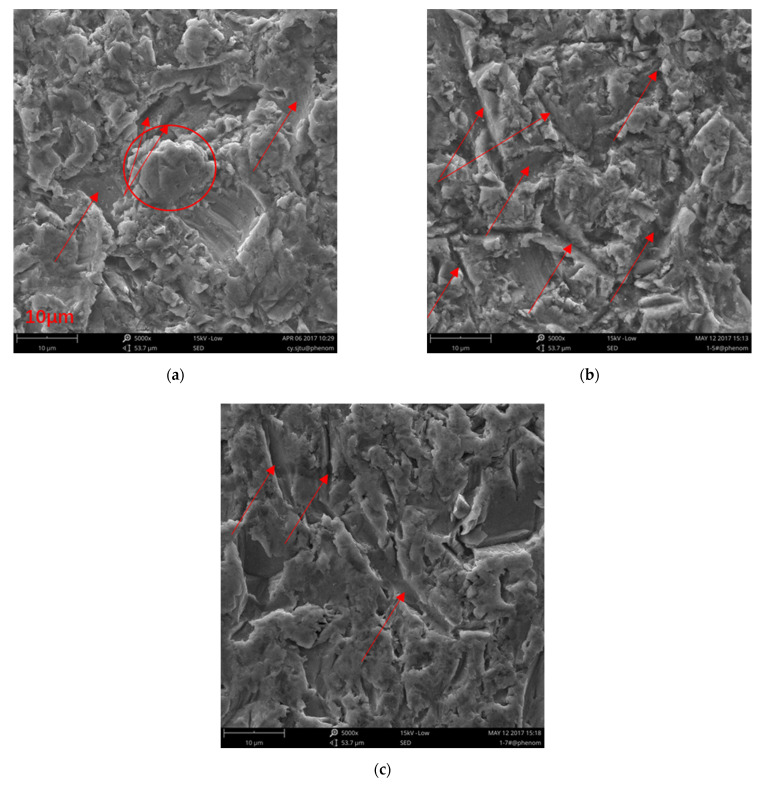
SEM images showing the microscale morphologies of the seriously eroded areas of the hydrophobic titanium surfaces eroded under different erosion angles. (**a**) 30°; (**b**) 60°; (**c**) 90°.

Figure 16 shows the microscale morphologies of the seriously eroded areas of the hydrophobic titanium surfaces under different impact angles. Although the microstructures were damaged, the images still provide useful information to understand the erosion mechanisms responsible for material loss, which differed based on the impact angle. 

In Figure 16a, a piece of material with a size of approximately 10 μm (indicated by a red circle) is about to peel from the surface, leading to the formation of a deep crater. This resulted from multiple impacts and subsequent crack growth. This piece of material is liberated from the surface upon additional particle impacts. The ploughs in Figure 16a are not obvious because of the low impact angle, which indicates a lower normal impact force compared to higher impact angles. The material near the ploughs was removed by the cutting force, and only a few ploughs are observed on the surface. 

In contrast, in Figure 16b, many deep ploughs can be observed as a result of the high normal impact force. Compared to the 30° impact angle, less material loss occurred on the surface under the 60° impact angle. This is because the higher normal impact force led to plastic deformation and only part of material loss from the surface. Thus, the erosion rate was lower than for the 30° impact angle.

The surface shown in Figure 16c is smoother than the surfaces in Figure 16a,b, and the number and depth of ploughs are much lower. However, nearly the entire surface in Figure 16c is covered by a thin layer of plastic deformation material due to the high normal impact force. However, the material is not be removed until several adjacent cracks grow and become coherent.

**Figure 17:**
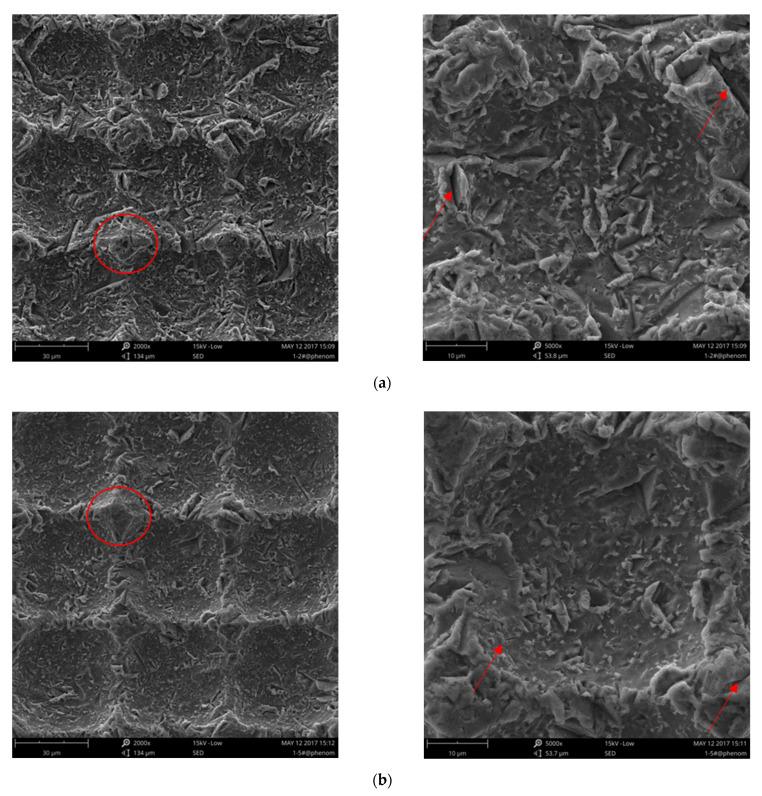

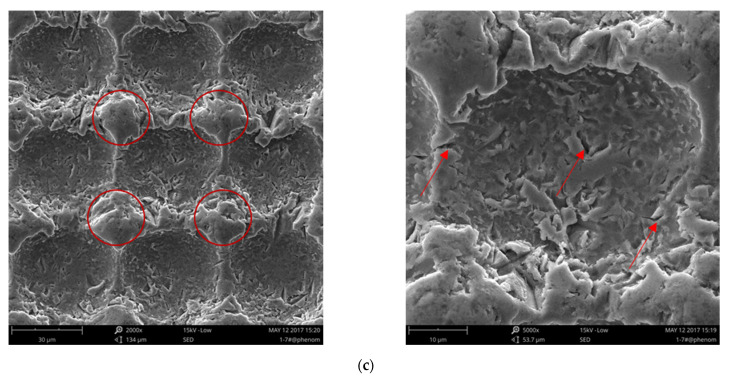
Microscale morphologies of the transition areas on the hydrophobic titanium surfaces under different impact angles. (**a**) 30°; (**b**) 60°; (**c**) 90°.

Figure 17 shows the microscale morphologies of the transition areas on the hydrophobic titanium surfaces under different impact angles. The microstructures of the samples shown in Figure 17 are only partly damaged, and the erosion mechanism responsible for the damage can be assessed based on the SEM images.

The failure modes of the microstructures differed based on the impact angle. At the low impact angle (30°; Figure 17a), a large component of the impact force was parallel to the surface, and the microstructures between the pits bore the impact force. The bending moment was sufficiently high to generate microcracks at the root of the structure. Many narrow ploughs can also be observed on the surface, as indicated by the red arrows in Figure 17a. These ploughs were formed via cutting by alumina erosive particles. In contrast, for the impact angle of 60° (Figure 17b), the component of the impact force parallel to the surface was smaller than that at 30°, while the component vertical to the surface was larger. This led to a lower number of ploughs and microcracks (marked by red arrows) along with some plastic deformation (marked by red circles). Thus, the erosion rate was lower for the impact angle of 60° compared to 30°. 

The hydrophobic titanium surface under a 90° impact angle (Figure 17c) showed obvious plastic deformation on the tops of the microstructures (marked by red circles). This was attributed to the high-speed normal impact, as in a “forge” process. The top of the structure was squeezed, forming a lip around the impact site. Subsequent impacts on the lip then caused the material of the sheet to break off the surface. For the impact deviated from the top of the microstructures, the impact force has a component parallel to the surface of the specimen. This causes a high level of tension stress and creates microcracks, as illustrated in Figure 17c. The microcracks continue to grow under continued impact loading. If two or more microcracks join together, material loss occurs.

## 4. Conclusions

Hydrophobic micro/nanostructured titanium surfaces have potential applications in de-icing and anti-icing systems. However, their endurance under high-speed erosion conditions remains a major concern. In this study, the erosion mechanisms of hydrophobic micro/nanostructured titanium surfaces were experimentally studied. The test rig and method developed in this paper provide a different and more severe way to study the durability of the micro/nanostructured titanium surfaces than that in ref. [19,20,23]. The main conclusions are summarized as follows:

(1) Cubic pit microstructures with small depth-to-width ratios fabricated by photoetching can improve the hydrophobicity of titanium alloy surfaces. The contact angle of smooth titanium alloy increased from 60.2° to 114.6° after photoetching. Photoetching can be used to conveniently produce hydrophobic surfaces with low cost and low residual stress, making it a good candidate for generating anti-icing and de-icing coatings.
(2) A high-speed sand erosion rig was designed and calibrated to conduct high-speed erosion tests of the micro/nanostructured titanium surfaces. The erosion rate of the micro/nanostructured titanium surface depended strongly on the erosion angle and speed, and the relationship was the same as that observed for bulk titanium alloy.
(3) Under the same impact conditions, the erosion rate of the micro/nanostructured titanium surface was similar to that of the smooth titanium alloy, indicating that the hydrophobic surface fabricated on the bulk material had erosion-resistant capability.
(4) The material loss mechanisms of the micro/nanostructures under different impact angles were compared. The findings provide useful information for the optimization of micro/nanostructures with the goal of improving erosion resistance.

The presented results provide useful information for the optimization of micro/nanostructures with the goal of improving erosion resistance. In future work, we will conduct in-depth research on the durability of micro-nanostructures coated with low surface energy hydrophobic materials, compare the advantages and disadvantages of different surface treatment methods, and seek the processing methods to improve the durability.

## Figures and Tables

**Table 1:** Major research advances.

Researchers	Main Research Content
Ghalmi et al. [13]	The effects of polytetrafluoroethylene (PTFE) nanostructural roughness on ice adhesion strength.
Susoff et al. [14]	Adhesion strengths between different coatings and ice, and the effect of surface roughness.
Fortin et al. [15]	Development of the centrifuge adhesion test to measure the icephobicities of icephobic coatings.
Kreeger et al. [16]	Basic requirements for icephobic coating and material.
Caroline et al. [17]	Properties of icephobic coatings for aerospace applications.
Golovin et al. [19]	The mechanisms for the extremely low ice adhesion between coatings and ice.
Cerro et al. [20]	The anti-ice properties of plasma-deposited hard coatings in combination with laser-machined patterns.
Zhang et al. [23]	Droplet erosion durability of a commercially available superhydrophobic coating.

**Table 2:** Design parameters of the erosion test rig.

Parameter	Units	Value
Impact speed	m/s	30–250
Impact angle	°	20, 30, 60, 90
Nozzle diameter	mm	4.8
Specimen diameter	mm	76.2
Hopper volume	liter	1.5

## Data Availability

All data supporting this study are available in the article.
